# Conservation Planning with Uncertain Climate Change Projections

**DOI:** 10.1371/journal.pone.0053315

**Published:** 2013-02-06

**Authors:** Heini Kujala, Atte Moilanen, Miguel B. Araújo, Mar Cabeza

**Affiliations:** 1 Metapopulation Research Group, Department of Biosciences, University of Helsinki, Helsinki, Finland; 2 Department of Biodiversity and Evolutionary Biology, National Museum of Natural Sciences, Consejo Superior de Investigaciones Científicas, Madrid, Spain; 3 ‘Rui Nabeiro’ Biodiversity Chair, Centro de Investigação em Biodiversidade e Recursos Genéticos, University of Évora, Évora, Portugal; 4 Center for Macroecology, Evolution and Climate, Department of Biology, University of Copenhagen, Copenhagen, Denmark; CNRS, University of Montpellier II, France

## Abstract

Climate change is affecting biodiversity worldwide, but conservation responses are constrained by considerable uncertainty regarding the magnitude, rate and ecological consequences of expected climate change. Here we propose a framework to account for several sources of uncertainty in conservation prioritization. Within this framework we account for uncertainties arising from (i) species distributions that shift following climate change, (ii) basic connectivity requirements of species, (iii) alternative climate change scenarios and their impacts, (iv) in the modelling of species distributions, and (v) different levels of confidence about present and future. When future impacts of climate change are uncertain, robustness of decision-making can be improved by quantifying the risks and trade-offs associated with climate scenarios. Sensible prioritization that accounts simultaneously for the present and potential future distributions of species is achievable without overly jeopardising present-day conservation values. Doing so requires systematic treatment of uncertainties and testing of the sensitivity of results to assumptions about climate. We illustrate the proposed framework by identifying priority areas for amphibians and reptiles in Europe.

## Introduction

Observed increases in global average temperatures, rise in the global average sea level and changing patterns and frequencies of extreme weather events, strongly suggest that the climate is changing according to model predictions [1]. Ecosystems are already responding to such changes, with species range shifts, phenological changes, and changes in species’ abundances and community composition [1], [2]. Climate change is thus recognized as a major threat to biodiversity and a big challenge to conservation [3]–[5].

Protected areas are critical instruments for safeguarding biodiversity. However, due to their increasing isolation in a matrix of highly modified landscapes, and the fact that they are geographically fixed, present-day protected areas are unlikely to be sufficient to accommodate and buffer climate-induced changes in biota [5]–[7]. Changes of species composition in protected areas are already being observed (e.g. [8]), and forecasts for the near future estimate that even bigger changes could take place [9]–[13]. In the past, conservation planning has primarily focused on preserving existing biodiversity pattern and has acted reactively with respect to new threats [14], [15]. But the need for a paradigm shift is being emphasized [5], [16]–[19]. Proactive responses to conservation challenges require the existence of reliable forecasts and a combination of present and future conservation goals, while limited conservation resources entail that these goals are to be met efficiently. The discipline of systematic conservation planning has thus seen the development of methods for solving such non-trivial conservation resource allocation problems, factoring in predicted species range shifts by modelling expected responses of species to climate change [5], [19]–[21]. Yet, an issue of concern is the uncertainty associated with both climate change and the consequent species responses [22]. This is particularly troublesome, because decision makers might be reluctant to base their conservation decisions on highly uncertain forecasts of future impacts that require trading-off scarce resources needed for mitigation of present day threats [19], [23].

An inherent source of uncertainty is the fact that we can only make projections about the future that are conditional to our knowledge and simplified model assumptions. To understand the potential impacts of future climate change scientists have been forced to analyse responses against distinct but equally likely scenarios of future development. Scenarios are plausible combinations of circumstances used to describe a future set of conditions, such as the widely used SRES scenarios [24], [25] that consider a wide range of possibilities for changes in population, economic growth, technological development, improvements in energy efficiency, and the like. Scenario assessments are important for policymaking, outlining a potential range of outcomes and thus influencing decisions. But despite the pressing interest in scenario probability [26], [27], scenarios typically lack an associated likelihood, making it difficult to assess the relative risks of particular adaptation policies.

Perhaps the most widely discussed aspect within the climate change conservation context is the uncertainty that arises from different modelling approaches when forecasting species distribution shifts. Most often effects of climate change on species distributions are predicted using niche models (also called bioclimatic envelope models, habitat models, or species distribution models). These are correlative approaches that relate current species occurrences to aspects of the environment, e.g., climatic variables, to then infer the sets of conditions in which species can be present. Ecological niche models include many assumptions and limitations: They are not concerned with dispersal processes, the dynamics of population at the leading or retracting edge, or the potential for adaptation or species’ interactions [28] (but see e.g. [29] and [30]). A large number of modelling approaches is available, and differences in their algorithms, parameterizations and the assumptions they make regarding the data often result in variation in outputs [31], [32]. Recent studies show that the variability in the predictions can be as high (or even higher) between different niche-based models than between different emission scenarios [33]–[36]. To cope with such inter-model variation, [37] advocate the use of multiple models within an ensemble forecasting framework. This approach allows identification of consensus among all forecasts, or consensus among subsets of forecasts, as well as exploration of the full breadth of inter-model variability.

Conservation exercises that account for climate change impacts frequently neglect the point that knowledge about the past and present is much more certain and accurate than knowledge about future. Thus one should give less weight to future projections than to present ones, especially if protecting important future locations comes at the expense of protecting important locations at present. Despite noticing that inter-model variability increases with time, existing conservation planning approaches have implicitly treated both current and projected distributions as equally certain (e.g. [20], [21], [38]). Here we suggest a practical approach to conservation planning where several sources of uncertainty, including alternative emission scenarios, can be accounted for. We demonstrate the proposed approach by identifying conservation priorities for European amphibians and reptiles. We first demonstrate how inter-model variability is spatially distributed across the region and how this can be accounted for in conservation prioritization. We then look at differences that rise from planning with different SRES scenarios, and identify priority areas that are common across all scenarios. We also assess the potential risks of planning conservation areas with a ‘wrong’ scenario. Finally, we evaluate trade-offs between conserving the present vs. conserving the future when giving increasing weight to future distributions in relation to present distributions.

## Materials and Methods

Both present (hereafter referred as ‘baseline’) and future projections of species potential distributions are based on the results published in [39] and available for download at http://www.ibiochange.mncn.csic.es/projects/former-projects/alarm/outputs/data. These models were fitted for 42 amphibian and 64 reptile species using species distribution data derived from the Atlas of amphibians and reptiles in Europe [40] and climate parameters derived from [41]. Five different climate parameters for the period 1961–1991 (referred to as ‘baseline data’) were used. Variables included mean annual temperature (°C), mean temperature of the coldest month (°C), mean temperature of the warmest month (°C), mean annual summed precipitation (mm), and mean sum of precipitation between July and September (mm). The future projected values for the climate variables were derived with the HadCM3 (Hadley Centre for Climate Prediction and Research’s General Circulation Model) [41] climate change model following four IPCC SRES storylines (A1F, A2, B1, B2) [42].

**Figure 1:**
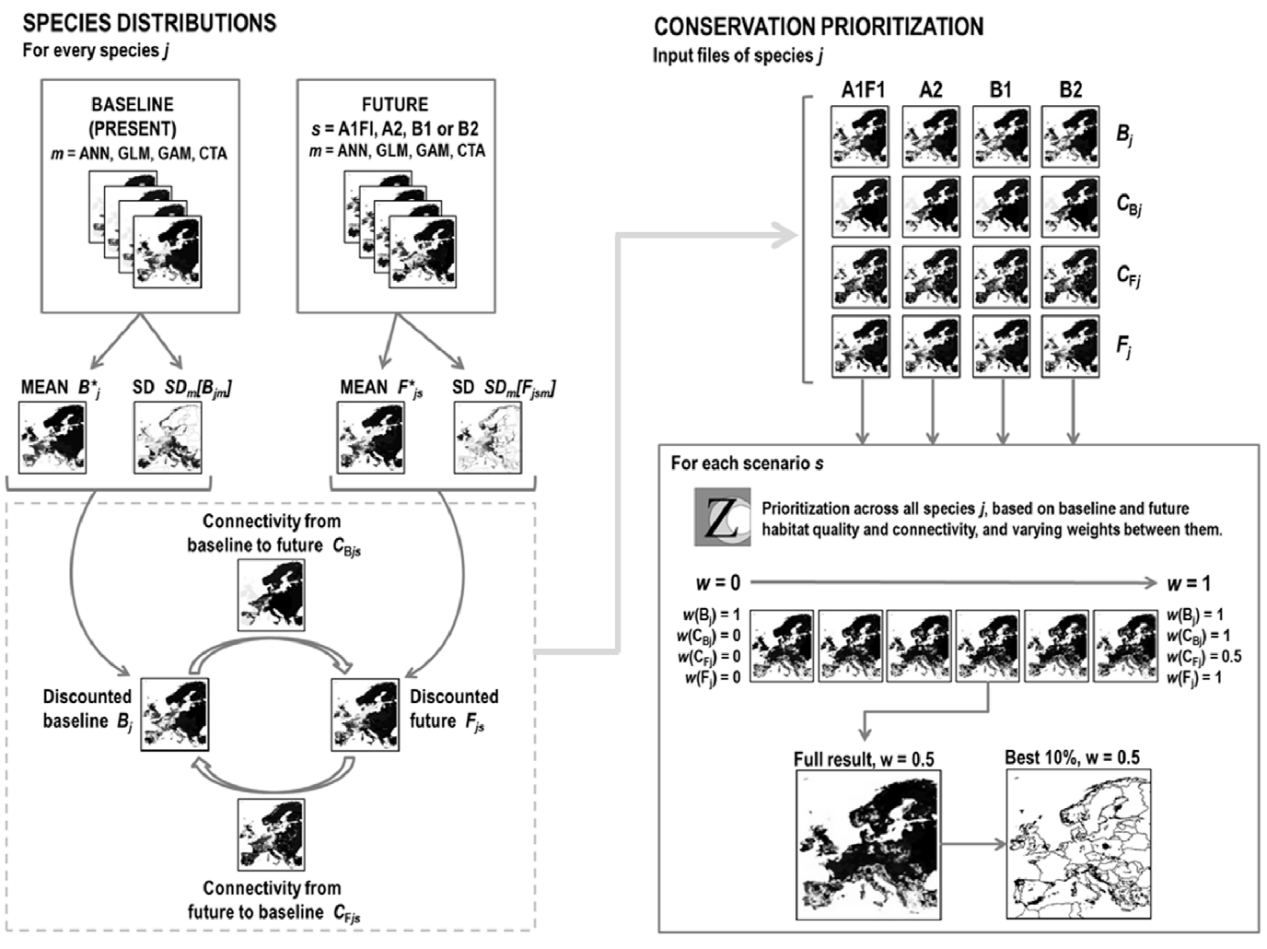
A flow chart showing how species distribution maps for baseline and future are created across the different modelling techniques *m*, discounting the mean values (denoted by an asterix) with inter-model variability (standard deviation, see text). Connectivity maps *C_Bj_* and *C_Fj_* are created based on the discounted baseline and future layers, resulting in four different input maps per species *j* and per scenario *s*. Prioritization is done separately for each scenario, producing multiple results per scenario where weights given to future distributions and connectivities are varied. Trade-off curves (Fig. 5) and comparisons between scenarios (Fig. 3) are done by focusing on the top 10% priorities of each Zonation result.

In [39] potential distributions at 10′ (ca. 10–16 km) grid cells were modelled with four niche-based modelling techniques (artificial neural networks [43], generalized linear models [44], generalized additive models [45], and classification tree analyses [46]) and distributions were projected into the future (2080) using an earlier S-Plus version of the well-known modelling BIOMOD package in R [47]. In our analysis we filtered the modelled probabilities of occurrence for the baseline period with observational data [40] so that values for cells from which no observations have been made for species were set to zero [9]. For each species we obtain four baseline distributions (*B_m_*) and 16 future distributions (*F_m,s_*), *m* denoting the type of bioclimatic model (see above) and *s* denoting the SRES scenario (Fig. 1).

### Conservation Priority Setting

We implemented a spatial conservation prioritization procedure that accounted for (i) multiple species, (ii) their estimated local probability of occurrence both for the baseline and future periods, (iii) basic connectivity requirements of the species, (iv) alternative climate change scenarios and their impacts on species, (v) uncertainty in the modelling of species potential distributions, and (vi) different levels of confidence about the baseline and future modelled distributions as well as the influences of connectivity.

We implemented the conservation prioritization analysis using the Zonation framework and software [48], [49], which is particularly well suited for the analysis of large GIS-based raster grid data sets that describe the distributions of many biodiversity features, such as species, habitats or ecosystem services [50]–[52]. Zonation does not use *a-priori* defined conservation targets. Rather, it produces a hierarchical priority ranking across all grid cells in the landscape based on occurrence levels and connectivities for species in cells, while balancing the solution simultaneously for all species used in the analysis [48], [53]. The areas of highest priority across species can then be identified simply by taking any given amount of area with highest priority ranks. After a top area has been selected, it is possible to calculate from original distribution data how large a proportion of each species’ entire distribution is captured by that selection. Below, we explain how connectivity and uncertainties were accounted for using the features available in the publicly available Zonation software, versions 2.0 and later [49] (www.helsinki.fi/bioscience/consplan/). We used the Core Area Zonation variant, which favours selection of high-quality areas for all conservation features even when they occur in relatively feature-poor areas [48], [54].

The primary units in our analysis were the modelled baseline and future species distributions. Connectivity from the baseline situation to the future was implemented via the ecological interactions (type 1) technique of Zonation (called “species interactions” in version 2.0), which allows calculation of connectivity between two distributions [55], [56]. This technique weights the local quality of one distribution by metapopulation-type connectivity to another distribution, either between species or, as in this case, between baseline and future distributions of the same species. We used two connectivity distributions per species, one for the connectivity from the baseline to the future and another for connectivity back from the future to baseline (Fig. 1). The former of these distributions represents source areas from where dispersal to future distribution areas is expected. The latter represents stepping-stones, which are expected to help species reach the core areas of their future distributions. The spatial scale of the interaction (here connectivity) is set by a species-specific parameter *β_j_*, which scales the mean decay distance of a two-dimensional negative-exponential dispersal kernel. Amphibians and reptiles are in general considered to be poor dispersers [57]–[59], although there are large variations in reported dispersal distances [57], [60]. As movement of amphibians is highly limited by their dependency on available water, and because both amphibians and reptiles are ectotherms characterized by strong home range fidelity, it has been speculated that species in these two groups will experience particularly large challenges in tracking climate change by dispersal [58]. Based on information retrieved from literature we set the parameter *β_j_* to correspond a conservative 0.2 km/year dispersal capability.

Thus, for one species we have four potentially relevant distributions to be covered in priority setting under each SRESS scenario. These are the baseline, future, dispersal source (connectivity from the baseline to the future) and stepping stone (connectivity from the future to the baseline) distributions, denoted by *B_jsm_*, *F_jsm_*, *C*_B*jsm*_ and *C*_F*jsm*_, respectively, where *j* is index for species, *s* is index for emission scenario and *m* is index for habitat modelling method (Fig. 1). In the full Zonation runs described in Figure 1 all four distributions for each species were prioritized simultaneously. We also looked at the relevant importance of each of the four distributions within the best 10% of the full run: this was achieved by repeating the prioritization across all species, but using only one type of distribution at a time (i.e. only present, only connectivity to present etc.) and cutting the resulting priority map with the top 10% priorities of the full run. Each grid cell within the top 10% priorities where then qualified as important present core, source, stepping stone or future core depending on which of the categories it received the highest priority ranking in.

Note that these quantities are matrices across the landscape; each distribution is a rectangular grid with the value *P* for each grid cell representing the predicted feature (species probability of occurrence or connectivity), conditional on the emission scenario and habitat model used. We produced an aggregate best prediction, P*_js_, for a species *j* in each grid cell as the mean across all different models, assuming emission scenario *s*. Using the future distribution as example, the aggregated best prediction would be F^*^_js_ = *E_m_*[*F_jsm_*], where *E_m_*[] represents expected value taken across all distribution models *m* (i.e. the mean). Another quantity used is the (again cell-specific) uncertainty in the prediction for species at any given location, measured as the standard deviation of the predictions across models, and denoted by *SD_m_*[*F_jsm_*].

### Accounting for Uncertainties

Throughout prioritization exercises we account for various sources of uncertainty and quantify their impacts in the following ways (see also Fig. 1):

1. Distribution discounting to account for variation between niche models. Distribution discounting penalizes predicted occurrence probabilities according to a measure of uncertainty associated with the prediction [61]. In the present application, a multiple of the standard deviation of predictions across models, *SD_m_*[*P_jsm_*], was subtracted from the mean prediction P^*^_j_, which represented our nominal best estimate. The subtraction is applied to each cell separately. Technically, we set *P_js_*(α) = max{0, P^*^_js_−α*SD_m_*[*P_jsm_*]}, where α is a parameter called the horizon of uncertainty in information-gap decision theory [62]. Our base-analysis used α = 1, corresponding to subtracting one standard deviation off the mean. With this procedure we give the highest value to those species occurrences *j* that have high mean probability and low standard deviation across all niche models. Distribution discounting is applied separately to the baseline and future distributions before calculating connectivity between baseline and future layers.
2. Impacts of different potential climate scenarios. Analyses are done separately for each emission scenario and solutions from different scenarios are compared using statistical tests. In particular, no averaging across emission scenarios was done at any point, because the scenarios are considered mutually exclusive. More specifically, by using a paired T-test we assess if conservation prioritizations based on different potential futures differ significantly in the amount of species present (baseline) and future distributions covered by the best 10% priority rank areas. In addition we undertake a risk and opportunity analysis by planning a conservation prioritization using one scenario and evaluating it against the others. This sensitivity analysis illustrates the potential situation where conservation planning has been done with wrong expectations of future impacts. We also explore which species were most adversely affected if conservation actions are based on false assumptions about the future development of climate change.
3. Weighting of baseline and future. Our third uncertainty approach is to weight the priority of baseline and future distributions of species differentially. This operation recognizes that knowledge about the future is more uncertain than the present, and that our understanding on the influence of connectivity is less certain than our understanding on the influence of habitat quality. This is because future predictions include major uncertainty about the degree of climate change that will happen, and on top of that the connectivity distributions include further uncertainty about species-specific dispersal and colonization distances. Hence, connectivity from future to present (*C_Fj_*) receives the lowest weight as it includes uncertainty about both species-specific connectivity features as well as future habitat quality upon which the connectivity calculations are based. Denoting by *w*(*D*) the weight given for a particular kind of a distribution *D*, qualitative considerations of uncertainty suggest that *w*(*B_j_*)>*w*(*C*_B*j*_) ≥ *w*(*F_j_*), *> w*(*C*_F*j*_). We use *w*(*B_j_*) = 1, and varying weights (0–1) for the future and connectivity layers scaled as follows: *w*(*C*_B*j*_) = *w*(*F_j_*) = 2x *w*(*C*_F*j*_).

We evaluate reductions and gains in conservation value for both the baseline and the future with increasing weight given to future distributions. When exploring the first two sources of uncertainty (points 1 and 2 above), we give equal weights to baseline and future distributions, hence following the above logic *w*(*B_j_*) = *w*(*C*_B*j*_) = *w*(*F_j_*) = 2x *w*(*C*_F*j*_).

## Results

**Figure 2:**
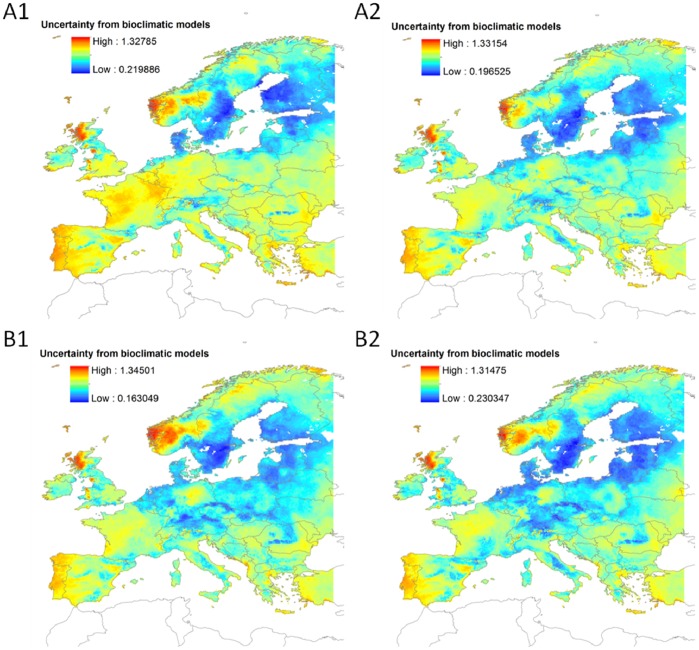
Areas of disagreement between models and across species, for scenarios A1, A2, B1, and B2, all for 2080. Uncertainty is illustrated as the per grid average across species of the coefficient of variation (ratio between the standard deviation and the mean) of predicted suitabilities by the four bioclimatic models. Orange areas indicate cells for which on average the standard deviation equals the mean.

Uncertainty due to the choice of niche model varied substantially across species (Fig. S1), between-model variation in the predicted probability of occurrences being notably larger for projected future distributions than for the baseline period. Despite this, we could identify spatially congruent regions of both high model agreement and disagreement across all species (Fig. 2). Using the distribution discounting procedure we were able to account for uncertainty in predictions and thus prioritize areas of highest quality and highest certainty (see Fig. S2 for an example with scenario A1).

For each SRESS scenario (A1, A2, B1 and B2) we produced a conservation prioritization that accounted simultaneously for species baseline distributions, future distributions, sources and stepping stones (*B_jsm_*, *F_jsm_*, *C*_B*jsm*_ and *C*_F*jsm*_). When species baseline and future distributions are weighted equally, the top 10% of all prioritizations cover a larger average proportion of species baseline distributions in comparison to future distributions (Table 1, diagonal and last column). Optimizing conservation for different SRES scenarios resulted in small but significant differences, both in the conservation level achieved for species baseline distributions (Paired T-test; p<0.001 in all comparisons); as for future distributions (Paired T-test, p<0.001 in all comparisons except between B1 and B2, p = 0.054, and between B1 and A2 p = 0.082). Scenario B2 resulted in highest overall conservation outcomes, where the top 10% of the landscape could potentially protect the highest average representation across species’ baseline and future distributions (Table 1). This is because present and future distributions are spatially most aggregated under scenario B2. Scenario A1, on the other hand, resulted in lowest conservation outcomes, both for baseline and future distributions, caused by a higher spatial spread of predictions for scenario A1. Optimizing conservation for different scenarios resulted in high differences in the expected protection of future distributions for some species. For example, almost 56% of the future distribution of the Iberian Rock Lizard (*Lacerta monticola*) could be protected when optimization was done with scenario B1, but only 27% if scenario A1 was used.

**Table 1:** Average representation level of baseline and future distributions, and expected conservation losses when planning is done for one scenario, but another scenario takes place.

Scenario that takes place		A1	A2	B1	B2	Baseline
Planned with	A1	*27.6*	**1.0** (16)**	**2.3** (18)***	**2.2** (16)***	*38.5*
	A2	**3.5** (60)***	*29.9*	**1.8** (17)***	**1.0** (4)***	*40.6*
	B1	**3.5** (67)**	**0.5** (22) ^ns^	*30.6*	**0.4** (11)**	*41.0*
	B2	**3.7** (66)***	**0.1** (20) ^ns^	**0.9** (21) ^ns^	*31.3*	*41.5*

The diagonal, in italics, shows the average proportion of future distributions of species represented within the top 10% priority sites when planning is done for a correct scenario (e.g. we plan for scenario A1 and scenario A1 takes place). Outside the diagonal, bold numbers indicate the average percentage loss in protection of future distributions that species experience when planning is done for one scenario, but another scenario takes place. Numbers in brackets show the maximum individual loss across the species pool, and stars indicate significance level for pair-wise comparisons across all species (Paired T-test, ***p<0.001;**p<0.01; *p<0.05; ^ns^non-significant). Last column shows the average proportion of species baseline distributions protected, when prioritization is done with each of the four SRES scenarios. In all solutions baseline and future distributions are weighted equally.

**Figure 3:**
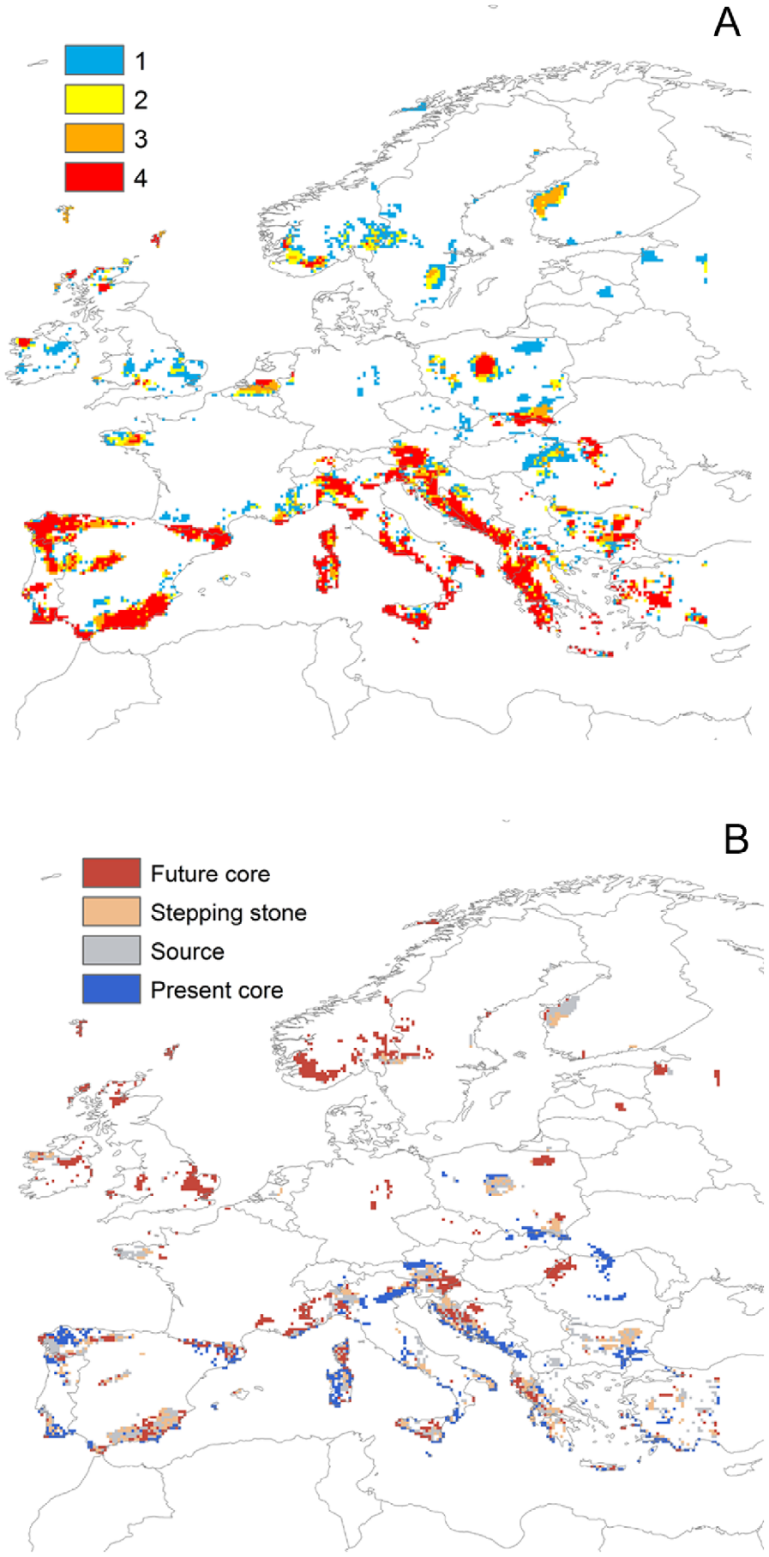
Top 10% conservation priorities for European amphibian and reptiles, including baseline distributions, projected future distributions and connectivity between them, when equal weight is given to present and future. A) Overlap of priorities across the four SRESS scenarios. Red indicates areas identified as top 10% priorities with all four scenarios; blue areas are identified by only one scenario B) Classification of the top 10% priorities into their relative importance as baseline cores, future cores, sources and stepping stones. Baseline and future cores were identified as the areas from the top ranked cells that according to habitat quality would be most important for species within their present and future distributions. Sources indicate areas that are most important for dispersal from present to future areas as climate changes. Similarly, stepping stones facilitate species migration to future core areas. They are parts of the predicted future distribution best connected to the present distribution.

Despite these differences, the spatial overlap between the conservation priorities identified for any two scenarios was high, 70–84%, being the highest between scenarios A2 and B2 (Fig. 3a). These common top priority areas could be classified into their relative roles as baseline and future cores, sources and stepping stones (Fig. 3b). Areas identified as highly important baseline cores with the four scenarios tended to be located in the Mediterranean region and in Eastern Europe, whereas the majority of the important future core areas were located in northerly parts of Europe (e.g. Scandinavia, Great-Britain and Ireland). Sources or stepping stones coincided for many species due to overlapping baseline and future distributions and the relatively short estimated dispersal distances. Overall, the solutions for the four scenarios shared 42% of the top 10% priority cells (Fig. 3a). Together, these common priorities covering 6.4% of the region could protect 30.7% for the baseline period and 12.3%, 14.9%, 15.6% and 16.2% for scenarios A1, A2, B1 and B2 correspondingly. Notably, over 30% of the cells were identified as priority in a single scenario.

**Figure 4:**
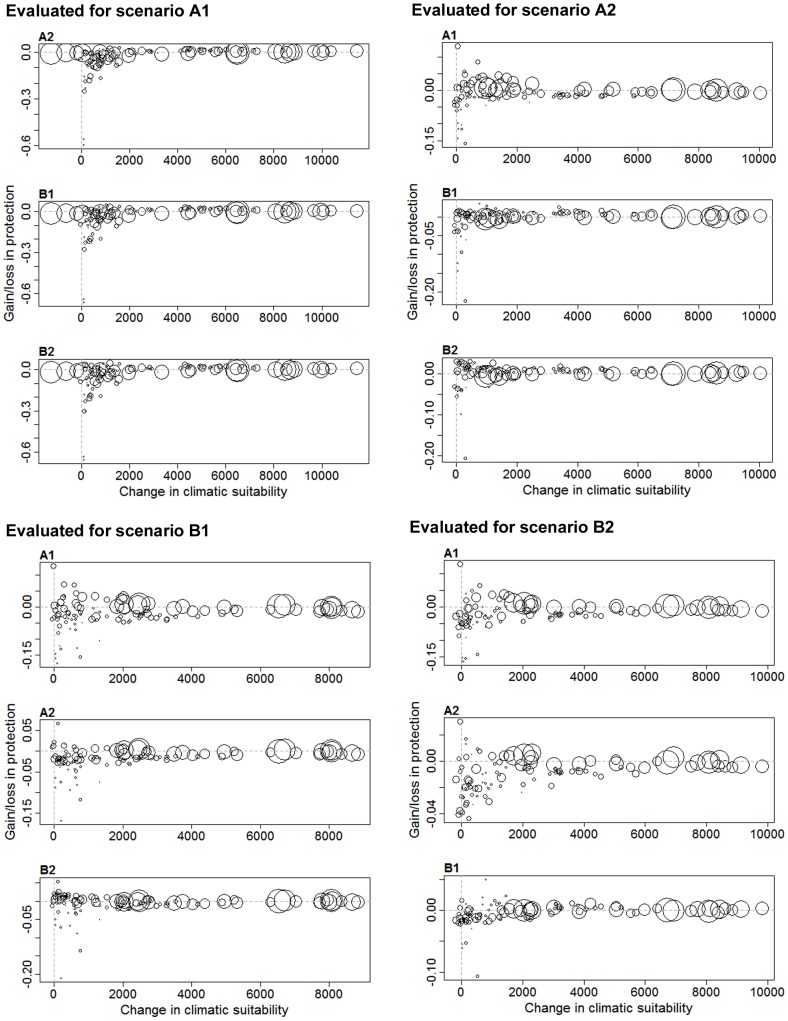
Relative losses and gains for a combination of planning and evaluation scenarios, indicated by negative (loss) and positive (gain) values in the y axis. Circles are species and circle size reflects the sum of baseline climatic suitability across cells. Panels A–D correspond to evaluation scenarios A1, A2, B1, and B2 respectively. Graphs within these panels correspond to the planning scenarios. Species are distributed along the x axis according to the expected change in future climatic suitability according to the evaluation scenario. Negative values in the x axis indicate species expected to experience a decrease in future climatic suitability.

Next we evaluated what are the gains or losses when we optimise conservation priorities assuming one scenario, but then another scenario takes place. This cross evaluation revealed notable losses in expected protection of up to 60% for some species. Largest reductions in average expected protection occurred if scenario A1 took place, but planning was done for any other scenario (Table 1; Fig.4). Similarly, a larger number of positive surprises [61] in the average coverage are expected when planning is done for A1 but other scenarios take place. However, note that these positive surprises, as measured in terms of average coverage of distributions, come with the cost of not achieving a balanced conservation solution across all species (Table S1). This can be seen in the variation of expected losses and gains across species (Fig. 4). Both the baseline extent of climatic suitability and the expected contraction or expansion of this suitability can partially explain these differences (Spearman’s rank correlations for the significant cases: rho = 0.4–0.66, p<0.001 for total baseline suitability, and rho = 0.25–0.63, p = <0.001–0.03 for expected contraction/expansion; Table S2), with a tendency for large-range and extensively expanding species to show smaller differences. Species such as Pygmy Algyroides (*Algyroides fitzingeri*) and Tyrrhenian Wall Lizard (*Podarcis tiliguerta*) with small baseline distributions and even smaller future distributions were the ones for which planning for the wrong scenario made largest negative differences.

**Figure 5:**
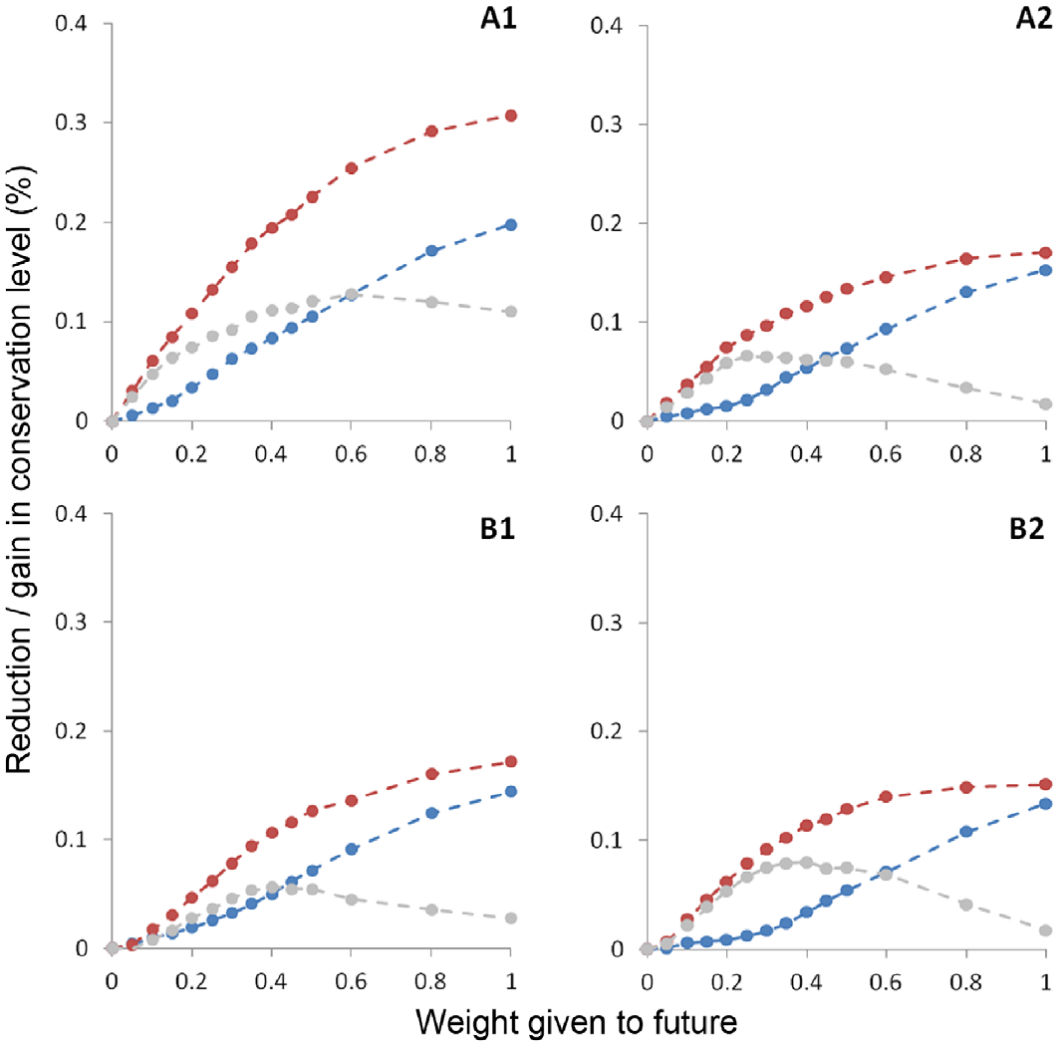
Trade-off curves for reductions in baseline conservation level (blue) and gains in future conservation level (red), as higher weight is given to the future, for the 4 SRESS scenarios. Each point corresponds to a different set of spatial priorities, selected with a different combination of weights for baseline and future layers. Baseline always receives a weight of one, while weight for the future is varied from zero to one. The difference between gain and loss curves is indicated with a grey line.

Exploration of the trade-offs between conserving the future and conserving the baseline revealed interesting patterns. Figure 5 shows the relative reductions in coverage of species baseline distributions and the relative gains in the coverage of species future distributions, when increasing weight is given to the future. Both gains and losses were largest for scenarios A1. The trade-off curves indicate that future layers should preferably be assigned a weight below one (i.e. larger priority to the baseline than the future) across all scenarios. Gain-loss differences (Fig. 5, grey line) peak at weights 0.25–0.4 for scenarios A2, B1 and B2, and at weight of 0.6 for scenario A1. Scenario A1 exhibited an interesting trade-off curve, where gains in future protection increase rapidly with small weights without significant reductions of baseline conservation, and the gain-loss difference remains highly positive even at high weights and large sacrifices in baseline conservation. In contrast, the curve for A2 flattens early, reflecting that increasing the weight given to the future results in quickly increasing baseline losses with only minor gains for future conservation achievements. Risk-averse planners might prefer to approach the problem of optimal weighting by choosing a maximum tolerable reduction of baseline protection level. A sacrifice of at most 5% of the baseline protection level implies different weights between 0.26 (A1) and 0.48 (B2) and result in gains between 11 and 14% (for scenarios B1 and A1, respectively) (Table 2).

**Table 2:** Comparison of conservation outcomes with risk averse weighting.

Scenario that takes place		A1	A2	B1	B2	Weight given to future
Planned with	A1	*22.9 (1.7)*	**26.7** (2.6)	**26.6** (2.5)	**27.9** (3.0)	0.26
	A2	**21.6** (1.7)	*28.1 (2.5)*	**27.7** (3.6)	**29.5** (2.9)	0.38
	B1	**21.9** (1.2)	**28.0** (2.2)	*28.6 (3.2)*	**29.7** (2.6)	0.4
	B2	**22.1** (1.0)	**28.5** (2.2)	**28.6** (3.3)	*30.5 (2.6)*	0.48

Here weight given to the future is determined with the trade-off curves (Fig. 4), by accepting a reduction of 5% in conservation of baseline distributions. The diagonal shows the average and minimum (in brackets) proportion of species future distributions represented in the top 10% priority sites, when planning is done with same scenario that eventually takes place. Numbers outside diagonal show the average and minimum representation of future distributions when planning is done for one scenario, but another takes place. Top 10% of all prioritizations achieve, on average, 45% representation of species baseline time period distributions, minimum representation across species being 4%.

## Discussion

In a crisis discipline like conservation biology [63], proactive efforts should take advantage of projected changes and offer solutions that enable decisions under inevitable levels of uncertainties. We have demonstrated a framework to deal with uncertainties, and illustrated it by identifying conservation priorities for amphibians and reptiles in Europe.

Reptiles and especially amphibians are threatened worldwide [64], [65] and recent studies implicate global warming as one of the causes of declines observed in these taxa [66]–[69]. Even so, niche-based projections of amphibian and reptile responses to warming in Europe are not overly alarming, with a great proportion of amphibian and reptile species projected to experience increases in climatic suitability across central and eastern Europe [39]. However, the degree to which the positive development in climatic suitability translates to distribution expansions is highly dependent on species dispersal abilities. [39] show that if all species are unable to disperse, then most species are in fact projected to lose range. Our models by the year 2080 indicate that the distributions of European amphibians and reptiles would change most drastically under the strong climate change scenario A1. Other scenarios show more moderate changes, future distributions under scenario B1 having the highest overlap with baseline distributions.

Dispersal is one of the largest sources of uncertainty in the context of climate change conservation. Yet most of the climate impact assessments are conducted with either a full dispersal or no dispersal (or both) assumptions. Only rarely plausible dispersal rates of the taxa under consideration are estimated and used (e.g. [21]). Here we showed how dispersal rates (whether species specific or common) can be technically efficiently accounted for, using a conservative estimate of dispersal which is in line with the published estimates for these taxonomic groups [57], [59], [70], [71]. Accounting for dispersal limitations in conservation planning is important, and we have shown here how species dispersal can be facilitated by identifying well connected sites between known present and potential future locations via the protection of sources and stepping stones.

We found that bioclimatic projections for the amphibians and reptiles of Europe varied significantly amongst the four niche models used. Yet we were able to account for this range of predictions in conservation prioritization by penalizing those locations displaying highest uncertainty with the distribution discounting procedure. Ensembles of forecasts, however, are often treated with common consensus approaches such as ensemble mean, weighted ensemble mean or ensemble median. Such metrics could be used similarly in the framework presented here, as could other values integrating information across several models – see [35]. The distribution discounting technique [61], which we used here, is a simple way of accounting for the first-order effects of uncertainty in pattern-based spatial conservation prioritization.

We have also illustrated the importance of trade-offs in securing both present and future representation of species. By giving varying weights to future sources, stepping stones and future core areas and exploring the resulting gain-loss curves we showed that compromises can be achieved without overly much jeopardising the conservation value achievable for baseline. Trading present for future or *vice versa* requires careful evaluation of the minimum coverage levels achieved for the present, as it is obvious that one cannot protect the future without having the species persisting through time, starting from the present. But determining what is a suitable level of coverage that allows persistence is not a trivial task. We found, however, that if conservation priorities are identified by looking at baseline distributions only, and ignoring future projections, some species may have little chances to persist in these locations because coverage of future distribution may become substantially reduced (a reduction of 30% on average if scenario A1 takes place). For example, the European Fire-Bellied Toad (*Bombina bombina*) is currently widespread but has been reported to have a declining population trend [72]. It would not have had any of its potential future distributions covered by our present baseline-only prioritization.

The large differences between the outcomes obtained when planning for different scenarios arise because baseline and future distributions overlap least in A1, and most in B1. Planning for various scenarios and evaluating overlap of priorities is recommended when searching for robust solutions, but we also emphasize the importance of a sensitivity analysis when doing scenario assessment, which helps identify both risks and potential for positive surprises. Different levels of risk-aversion in conservation planning, or conflicting policy agendas will then dictate what losses can be tolerated or how much weight is given to the possibility of positive surprises. For this particular example it at first appears as if worst conservation outcomes would follow planning for scenario A1, i.e. lower present and future coverage. However, a very risk-averse planner applying the precautionary principle may precisely chose to plan for A1, as priorities thus identified are expected to result in smallest negative surprises if some other climate scenario occurs. Having an adequate outcome for scenario A1 is harder than obtaining an adequate outcome for the other scenarios: in this sense planning for A1 can be seen as a risk-averse strategy. On the other hand, losses can be large if plans are based on any other scenario, but A1 takes place. Also, if the outcomes of these scenarios were to influence mitigation policies, it is clear that scenario A1 is the one we wish to avoid.

### Conclusions

Conservation planning in a changing world is a challenging task. Nonetheless studies evaluating the future performance of protected areas [11], [13], [73] call for the consideration of biodiversity on the move in conservation planning. Unfortunately, the magnitude of uncertainty in species’ bioclimatic modelling is currently so great that it might lead conservation planners, policy makers and other stakeholders to question the overall usefulness of science as an aid to plan for the near future. However, uncertainty is no excuse for inaction when the world is changing as rapidly as it is. We have presented a framework that encourages action despite uncertainties. Overall, we recommend the following process: the use of ensembles of forecasts to account for reasonable variation in projections; the exploration of uncertainties associated with socio-economic scenarios, and the evaluation of potential losses incurred if planning is done for the wrong scenario. When conservation planning resources are limited, and levels of protection for present-day biodiversity need to be traded-off against uncertain future extent of protection, it is necessary to explore such trade-offs and to identify solutions that minimally compromise present conservation for largest future benefits.

## Supporting Information

Figure S1 **Variation of uncertainty from niche models.** Variation of uncertainty is calculated using the mean of probabilities across all niche models and calculated for each species and grid cell. One standard deviation (SD) of the species-grid-specific mean is divided by the mean itself to reflect the magnitude of variation in each cell. These values are then averaged across cells for each species to produce boxplots of overall variation of uncertainty, presented here separately for present and each of the future scenarios.
(TIF)

Figure S2 **Impact of distribution discounting to spatial prioritization.** Panels A and B represent prioritizations done for future distributions under scenario A1 without (A) and with (B) discounting, respectively. The brown colors highlight the best 10% of the entire area. Areas of high uncertainty (C, red color) affect the prioritization result so that sites with high conservation value but large variation among niche models, and hence large uncertainty, are penalized by the discounting (red arrows in A and B). Note that some areas remain highly prioritized despite notable uncertainty (blue arrows). These are areas of high conservation value across all species, and although variation in predictions is high, the mean remains high after subtraction of the error.
(TIF)

Table S1 **Cross evaluation of conservation outcomes when planning is done with one scenario, but another takes place.** Numbers outside the diagonal show the percentage loss or gain in the expected average representation of future distributions in the top 10% priorities when planning is done with a wrong scenario. For example, if we plan conservation priorities based on scenario A1 (first row), and A1 actually takes place (first column), the top 10% priority sites will capture, on average, 27.6% of species A1 distributions (Table 1). But if scenario A2 takes place (first row, second column), the priority sites that were selected based on A1 will capture, on average, 4.3% more of species A2 distributions then what would be achieved if prioritization was done with A2. Note that this apparent gain in the protection of future sites comes with a cost of reduced protection in species baseline distributions. Here baseline and future distributions are weighted equally.
(DOCX)

Table S2 Correlation between expected conservation gain/loss of future distributions in the cross-evaluations and (A) extent of species current climatic suitability, or (B) expected contraction/expansion of climatic suitability. Expected change in climatic suitability is calculated between present and realized SRES scenario (i.e. scenario that takes place). Numbers show Spearman’s rank correlation coeffients and stars indicate significance level (***p<0.001; **p<0.01; *p<0.05; ^ns^non-significant).
(DOCX)
