# Supplementary figures and images for: Conservation Planning with Uncertain Climate Change Projections
Source: PLoS One. 2013 Feb 6;8(2):e53315. doi: 10.1371/journal.pone.0053315 (PMC3566137; doi:10.1371/journal.pone.0053315)

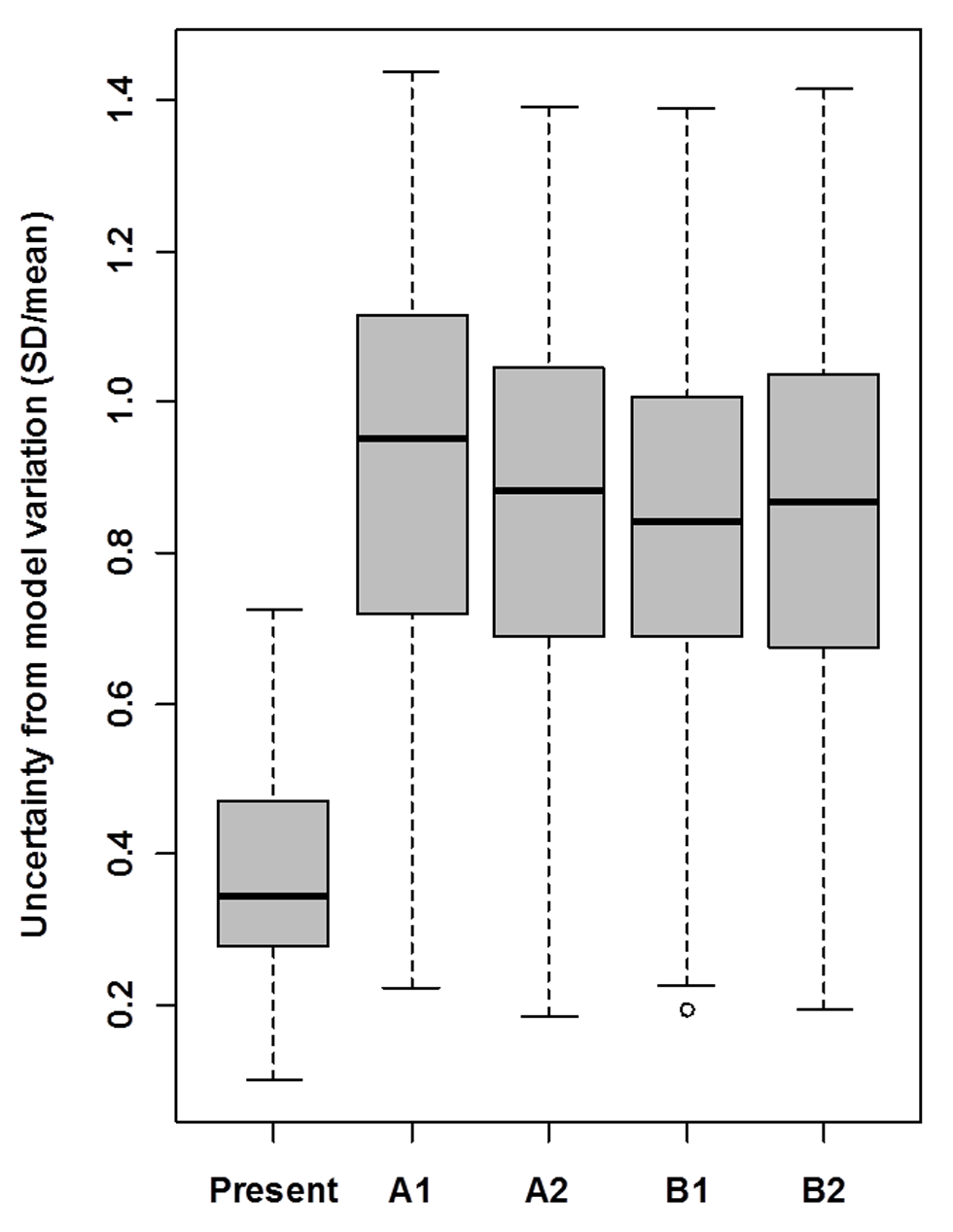

Supplement: Figure S1 — Variation of uncertainty from niche models. Variation of uncertainty is calculated using the mean of probabilities across all niche models and calculated for each species and grid cell. One standard deviation (SD) of the species-grid-specific mean is divided by the mean itself to reflect the magnitude of variation in each cell. These values are then averaged across cells for each species to produce boxplots of overall variation of uncertainty, presented here separately for present and each of the future scenarios. (TIF) [file pone.0053315.s001.tif]

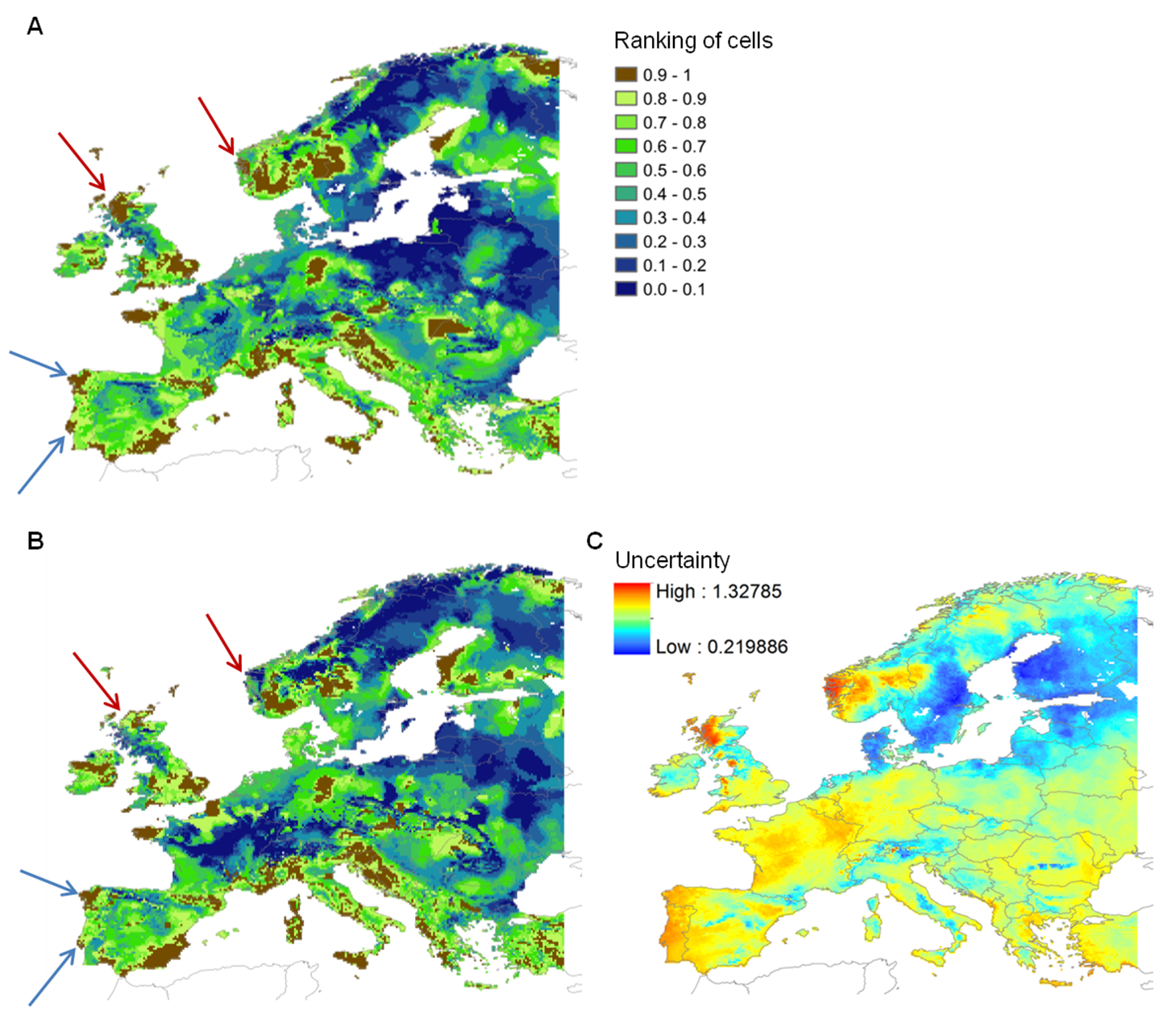

Supplement: Figure S2 — Impact of distribution discounting to spatial prioritization. Panels A and B represent prioritizations done for future distributions under scenario A1 without (A) and with (B) discounting, respectively. The brown colors highlight the best 10% of the entire area. Areas of high uncertainty (C, red color) affect the prioritization result so that sites with high conservation value but large variation among niche models, and hence large uncertainty, are penalized by the discounting (red arrows in A and B). Note that some areas remain highly prioritized despite notable uncertainty (blue arrows). These are areas of high conservation value across all species, and although variation in predictions is high, the mean remains high after subtraction of the error. (TIF) [file pone.0053315.s002.tif]
