# Supplementary material for: Conservation Planning with Uncertain Climate Change Projections
Source: PLoS One. 2013 Feb 6;8(2):e53315. doi: 10.1371/journal.pone.0053315 (PMC3566137; doi:10.1371/journal.pone.0053315)
Supplement: Table S1 — Cross evaluation of conservation outcomes when planning is done with one scenario, but another takes place. Numbers outside the diagonal show the percentage loss or gain in the expected average representation of future distributions in the top 10% priorities when planning is done with a wrong scenario. For example, if we plan conservation priorities based on scenario A1 (first row), and A1 actually takes place (first column), the top 10% priority sites will capture, on average, 27.6% of species A1 distributions (Table 1). But if scenario A2 takes place (first row, second column), the priority sites that were selected based on A1 will capture, on average, 4.3% more of species A2 distributions then what would be achieved if prioritization was done with A2. Note that this apparent gain in the protection of future sites comes with a cost of reduced protection in species baseline distributions. Here baseline and future distributions are weighted equally. (DOCX) [file pone.0053315.s003.docx]

**Table S1.**

| **Scenario that takes place** | | **A1** | **A2** | **B1** | **B2** | **Baseline** |
| --- | --- | --- | --- | --- | --- | --- |
| **Planned with** | **A1** | - | 4.3 | 2.6 | 5.4 | *38.5* |
|  | **A2** | -19.2 | - | -3.6 | 1.6 | *40.6* |
|  | **B1** | -21.3 | -4.0 | - | 0.9 | *41.0* |
|  | **B2** | -23.5 | -5.1 | -5.1 | - | *41.5* |
