# Supplementary material for: Conservation Planning with Uncertain Climate Change Projections
Source: PLoS One. 2013 Feb 6;8(2):e53315. doi: 10.1371/journal.pone.0053315 (PMC3566137; doi:10.1371/journal.pone.0053315)
Supplement: Table S2 — Correlation between expected conservation gain/loss of future distributions in the cross-evaluations and (A) extent of species current climatic suitability, or (B) expected contraction/expansion of climatic suitability. Expected change in climatic suitability is calculated between present and realized SRES scenario (i.e. scenario that takes place). Numbers show Spearman’s rank correlation coeffients and stars indicate significance level (***p<0.001; **p<0.01; *p<0.05; nsnon-significant). (DOCX) [file pone.0053315.s004.docx]

**Table S2.**

A.

| **Scenario that takes place** |  | **A1** | **A2** | **B1** | **B2** |
| --- | --- | --- | --- | --- | --- |
| **Planned with** | **A1** | - | 0.61 *** | 0.66 *** | 0.56 *** |
|  | **A2** | 0.02 ^ns^ | - | 0.58 *** | 0.45 *** |
|  | **B1** | -0.11 ^ns^ | -0.17 ^ns^ | - | -0.005 ^ns^ |
|  | **B2** | -0.03 ^ns^ | -0.11 ^ns^ | 0.40 *** | - |

B.

| **Scenario that takes place** |  | **A1** | **A2** | **B1** | **B2** |
| --- | --- | --- | --- | --- | --- |
| **Planned with** | **A1** | - | 0.21 * | 0.24 * | 0.43 *** |
|  | **A2** | 0.63 *** | - | 0.35 *** | 0.43 *** |
|  | **B1** | 0.52 *** | 0.25 ** | - | 0.48 *** |
|  | **B2** | 0.48 *** | 0.05 ^ns^ | -0.08 ^ns^ | - |
